# The Role of Imaging of Lymphatic System to Prevent Cancer Related Lymphedema

**DOI:** 10.3390/bioengineering10121407

**Published:** 2023-12-10

**Authors:** Vincenzo Cuccurullo, Marco Rapa, Barbara Catalfamo, Gianluca Gatta, Graziella Di Grezia, Giuseppe Lucio Cascini

**Affiliations:** 1 Department of Precision Medicine, Università della Campania “Luigi Vanvitelli”, 80131 Napoli, Italy; marco.rapa@me.com (M.R.); gianluca.gatta@unicampania.it (G.G.); 2 Nuclear Medicine Unit, Department of Diagnostic Imaging, Magna Graecia University of Catanzaro, 88100 Catanzaro, Italy; barbaracatalfamo@gmail.com (B.C.); cascini@unicz.it (G.L.C.); 3 Radiology Department, ASL Avellino, 83100 Avellino, Italy; graziella.digrezia@gmail.com

**Keywords:** lymphedema, imaging, lymphoscintigraphy, lymphangiography, MRL, ARM

## Abstract

Lymphedema is a progressive chronic condition affecting approximately 250 million people worldwide, a number that is currently underestimated. In Western countries, the most common form of lymphedema of the extremities is cancer-related and less radical surgical intervention is the main option to prevent it. Standardized protocols in the areas of diagnosis, staging and treatment are strongly required to address this issue. The aim of this study is to review the main diagnostic methods, comparing new emerging procedures to lymphoscintigraphy, considered as the golden standard to date. The roles of Magnetic Resonance Lymphangiography (MRL) or indocyanine green ICG lymphography are particularly reviewed in order to evaluate diagnostic accuracy, potential associations with lymphoscintigraphy, and future directions guided by AI protocols. The use of imaging to treat lymphedema has benefited from new techniques in the area of lymphatic vessels anatomy; these perspectives have become of value in many clinical scenarios to prevent cancer-related lymphedema.

## 1. Introduction

Lymphedema is characterized by the accumulation of protein-rich lymphatic fluid in the interstitium. Possible causes include congenital abnormalities, early and late primary conditions, and cancer-related or infective secondary lymphedema.

Cancer-related lymphedema ensues after the damage of lymphatic structures, as a consequence of lymph node surgical resection (axillary or inguinal) or direct tumor invasion.

The surgery for breast cancer treatment is the leading cause of cancer-associated lymphedema, because the estimated incidence after 3 years from sentinel lymph node dissection (SNLD) is 25%, rising to more than 30% after SNLD and radiotherapy [1].

Independent risk factors such as obesity, genetics and concomitant radiotherapy contribute to lymphedema development, along with the surgical technique and diagnostic methods used to plan it.

The coexistence of different underlying mechanisms may explain the delayed onset after surgery, irrespective of a clear clinical expression [2].

The clinical diagnosis is achieved by measuring differences between the limbs: in terms of volume, a difference higher than 100–200 cm^3^ is diagnostic; in terms of circumference, differences higher than 2 cm or an increase >10% are needed to recognize lymphedema [3].

These cut-offs are particularly effective in discriminating true lymphedema from simple differences in dominant and non-dominant arms, but potentially exclude mild forms of disease, even if symptomatic [3].

Many classifications are used to assess the severity of lymphedema; among them, the International Society of Lymphology (ISL) staging system is widely accepted [4].

Lymphedema is classified into four categories corresponding to subclinical (stage 0), mild (stage 1), moderate (stage 2) and severe (stage 3) according to symptoms and clinical presentation. Stage 0 is the earliest form presenting subjective symptoms as heaviness, tightness, fatigue and firmness in the absence of measurable swelling.

Stages 1 and 2 present measurable edema with spontaneous reversibility that improves with elevation or compression in stage 1, whereas irreversible edema irrespective of elevation or compression is seen in stage 2.

Moreover, the edema in stage 2 appears pitted, with indentations in early disease, but it evolves over time into a non-pitting pattern with the persistence of fluid stasis and subsequent fibrous changes. In stage 3, we see lymphostatic elephantiasis with the irreversible enlargement of limbs with severe fibrosis and skin changes such as acanthosis, fissures, ulceration, and rarely, lymphoangiosarcoma (Stewart–Treves Syndrome). This severe form is often complicated by recurrent episodes of infections/cellulitis. The ISL also defines arm measurements to correspond with the stage of lymphedema by changes in circumference from baseline. Stage 0 corresponds to no change in arm measurement. A change of sizes >5–10%, >10–20% and >20–40% corresponds to minimal, mild and moderate lymphedema, respectively. An increase >40% indicates severe disease.

Recent gradings have focused on quality of life rather than clinical manifestations, as reported in the Lymphedema Quality of Life Questionnaire for upper limbs (LYMQOL-UL) validated by Monticone et Al [5].

In recent years, new techniques of the study of the lymphatic system have been emerging with particular interest in sentinel lymph node mapping [6] and lymphedema diagnosis. The main features of the development process are the increase in spatial resolution, dose reduction and cost-efficacy.

The usage of imaging is becoming progressively wider these days to diagnose early forms of lymphedema and also to assess therapeutic intervention in advanced forms [7]. In recent years, two new techniques have shown promising results compared with the lymphoscintigraphic method, still the gold standard today, which are indocyanine green (ICG) fluorescence lymphangiography and magnetic resonance imaging (MRI) lymphangiography.

At first, valvular incompetence was considered an important pathophysiologic determinant of lymphedema, but it has since been confuted using ICG lymphography, which shows superficial lymphatic flow with high real-time quality. To prove this, Mackie et al. demonstrated that in a large series of patients, the incidence of retrograde flow from valvular incompetence was 3.7%, and just 0.3% of those had secondary cancer-related lymphedema.

## 2. Lymphoscintigraphy: An Overview of the Gold Standard Method

Scintigraphic studies of the lymphatic system began in the 1950s, and this continues to be the most widely used method in the world. The peculiar characteristics of radiocolloids make them ideal for visualizing a small vessel system without an autonomous pump system. The only forces that ensure lymphatic flow are hydrostatic and colloidal pressure [8].

There is a variety of radiopharmaceuticals used for studying the lymphatic system, not all of which are approved on different continents; for example, in Europe, the most widely used is ^99m^Tc albumin nanocolloid [9].

Aggregates of human albumin and other formulations differ in size, and thus have to be filtered to obtain dimensions suitable for specific purpose. The smaller aggregates (less than 100 nm in diameter) show higher uptake in the lymphatic district, resulting in better image quality, while larger ones (200–1000 nm) are progressively trapped in lymph nodes, allowing sentinel node mapping [10].

The surface charge of the particles as well as the binding to specific receptors exposed in the lymphatic system are other relevant aspects that affect radiopharmaceutical bio-distribution [11].

The subcutaneous injection of radioactive molecules results in optimal lymphatic representation as regards transport kinetics and time of persistence in the site [12]. In contrast, intradermal injections exhibit greater uptake into the blood vessels, decreasing the residence time of the radiocolloid by increasing lymphatic drainage flow. This kinetic aspect allows for better quantitative analysis than qualitative [13].

Lymphoscintigraphy, always performed on both limbs, can define the severity of lymphedema based on imaging findings, such as asymmetry in the lymphatic vessels, the presence of lymphatic collaterals, delayed lymph flow, the absence of uptake in regional lymph nodes and dermal backflow [14].

For an accurate description of the technical characteristics and interpretations of lymphoscintigraphic examinations, please refer to the Genoa protocol set out by the authors Villa G. and Campisi C. [15].

Moreover, subfascial injections may be used to study deep lymphatic drainage.

Therefore, the site of injection depends on the type of radiopharma and scope of imaging (e.g., sentinel lymph node mapping vs. lymphedema). Thus, all previously reported techniques are feasible and indicated for specific conditions.

To date, lymphoscintigraphy remains an extremely accurate test with high sensitivity (up to 96%) and very high specificity (up to 100%) in the diagnosis of lymphedema, as assessed by Hassanein H. et al. [16] in one of the largest case studies of 227 patients enrolled between 2009 and 2016, with similar results to other studies based on large case series, such as the one conducted by Gloviczki P. et al. [17].

Imaging-based and clinical classifications are difficult to compare, even if they are based on similar lymphoscintigraphic findings. Clear correlations between stages in lymphoscintigraphic classifications and patient-reported symptoms have never been demonstrated; moreover, the ISL staging system is suboptimal for patients referred to surgery lacking anatomical knowledge.

Among the various classifications, the Taiwan Lymphoscintigraphy Staging (TLS) is one of the clearest, and was recently proposed by Pappalardo M. and Cheng M. [18]. This is based on three scintigraphic features: visualization of proximal/intermediate lymph nodes, linear lymphatic ducts, and dermal backflow. According to these, the classification recognizes three patterns: normal drainage, partial obstruction, and total obstruction. The last two patterns are further divided into three stages. The TLS is reported in Table 1.

Over the years, thanks to microsurgical techniques applied to the lymphatic system such as lympho-venous anastomosis (LVA) and vascularized lymph nodes (VLN) for the treatment of lymphedematous pathology [20], lymphoscintigraphy has assumed a key role in planning surgical treatment.

Although there are no universally accepted classifications for this goal, Cheng M. et Al. proposed a comprehensive clinical imaging grading system called Cheng’s Lymphedema Grading System [19] that relates four parameters (circumferential difference (%), episodes of cellulitis (times/year), Taiwan Lymphoscintigraphy Staging and ICG lymphography) to the best possible treatment. The Cheng’s Lymphedema Grading System is reported in Table 2.

In relation to Cheng’s Lymphedema Grading System, for less severe cases, the most indicated treatment is complete decongestive therapy (CDT), which includes manual lymphatic drainage, band compression, exercise and skin care. Lympho-venous anastomosis (LVA) is performed in patients who do not want to wear elastic compression bands. For moderate–severe cases, a finer evaluation of the presence of functioning lymphatic ducts is mandatory, and only patients that demonstrate patent superficial lymphatic ducts are candidates for LVA. Vascularized lymph node transfer (VLN) is performed only if dermal backflow is present and the patients demonstrate nonfunctioning lymphatic ducts. In severe cases, the indication of VLN is always present, and association with additional surgical procedures such as liposuction or debulking surgery is often performed.

In severe cases, it is important to differentiate between obstructions in deep and superficial lymph vessels to establish multilevel surgical treatment [21]. However, it is not easy to assess differences with a planar method such as lymphoscintigraphy. For this purpose, methods such as SPECT, possibly associated with CT, have been used to improve spatial resolution, but without obtaining significant results [22].

Until now, however, there has been no close correlation between the results of pre- and post-operative lymphatic imaging methods and clinical objective findings, but in relation to the latter objective, based on preliminary studies on a small cohort of patients [23], it would appear that the lymphoscintigraphic method correlates the best with clinical and therapeutic outcomes.

### The Quantitative Method: Alternative or Complement to Gold Standard?

**Figure 1:**
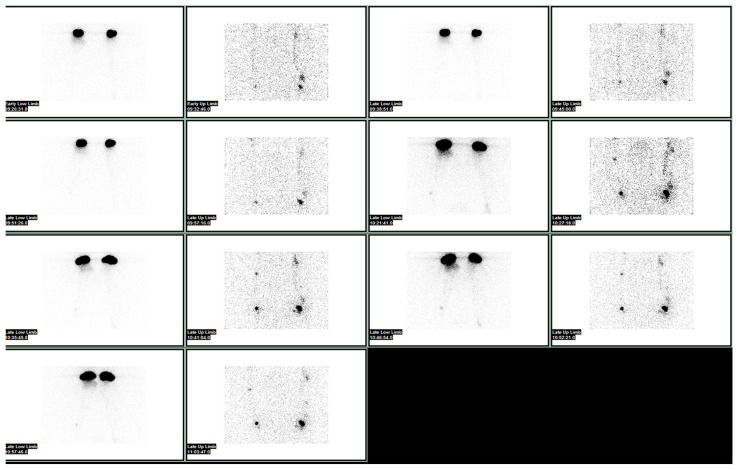
Normal lymphoscintigraphy of upper limbs performed after injection under the deep aponeurosis of the first interdigital spaces of hands bilaterally, and subsequently after a subdermal injection of the second, third and fourth interdigital spaces with ^99m^Tc albumin nanocolloid.

**Figure 2:**
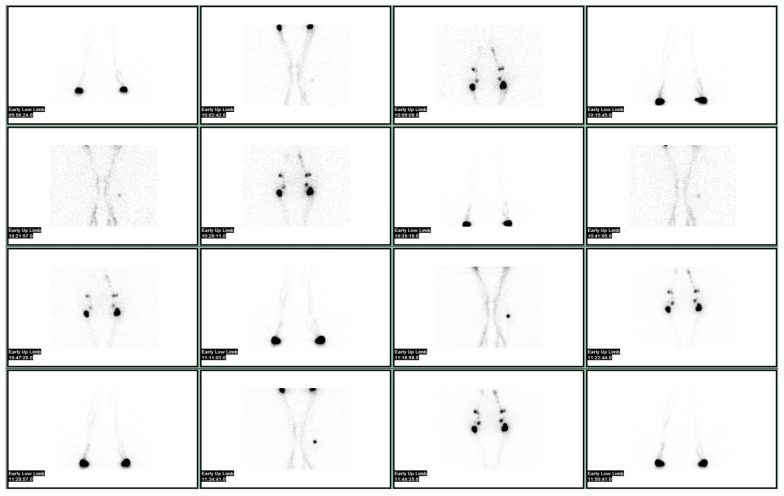
Normal lymphoscintigraphy of the lower limbs performed after the injection under the deep aponeurosis of the third finger of feet bilaterally, and subsequently after a subdermal injection of the second, third and fourth interdigital spaces with ^99m^Tc albumin nanocolloid.

Lymphoscintigraphy may be quantitative or qualitative. The differences in terms of uptake intensity as well as the transit time in proximal nodes, as well as the time for clearance and the time for appearance in blood, are some of the quantitative parameters that are measured (Figure 1 and Figure 2).

The quantitative method is more sensitive when used in the diagnosis of lymphedema, especially in the earliest stages, where slight differences are difficult to assess. The lack of standardized protocol and inconclusive results have negatively affected the use of quantitate parameters in clinical practice.

A recent study by Kwon et al. [24] has opened up the possibility of using quantitative parameters to predict surgical procedure outcomes. The aim of the study was to investigate factors predicting early and late treatment outcomes using lymphoscintigraphic factors before LVA.

The authors suggest that dermal backflow is a significant positive-predictive qualitative factor; moreover, the surgical effect is higher in patients with both proximal and distal dermal backflow. These finding may be related to the regurgitation of lymphatic fluids into subcutaneous tissues at high pressures due to the occlusion of lymphatic vessels. LVA restores lymphatic flow by reducing the pressures in the lymphatic district and producing significant clinical effects.

The authors have analyzed other quantitative parameters over different times (1 h and 2 h) such as lymph node uptake ratio, extremity uptake ratio and injection site clearance ratio. All of these have been correlated to the treatment response and to the volume difference ratio at 3 months and 1 year after surgery

The results between quantitative parameters and volume difference ratio, evaluated with Sperman’s rank correlation coefficient, were statistically significant only for extremity uptake ratio at 2 h (2 h EUR) in relation to volume difference ratio after 3 months (*p* = 0.016), and even more so at 1 year (*p* = 0.001). Regarding the relationship between quantitative parameters and therapeutic response, assessed by Mann–Whitney test, the results show statistical significance only for 2 h EUR in relation to therapeutic response at 1 year. In addition, patients with a high 2 h EUR showed greater volume reduction than patients with a low 2 h EUR (*p* = 0.027). All other quantitative parameters assessed at 1 and 2 h did not show statistical significance.

The study presents some limitations, such as the small group of patients (17) and non-homogeneity regarding the etiology of lymphedema (primary or secondary); however, it shows an advantage in terms of using both the dermal backflow pattern and extremity uptake ratio (EUR) at preoperative lymphoscintigraphy to predict therapy response in patients who will undergo to LVA. These results are in line with those of other similar studies.

At first, Yoo JN. et al. correlated quantitative and qualitative lymphoscintigraphic results to the arm circumferences in breast cancer patients with lymphedema secondary to ALD [25]. Here, 72 patients with cancer-related lymphedema were divided into three qualitative groups, defined as follows: normal pattern with normal lymphatic system, with the visualization of superficial lymphatic system and normal axillary lymph nodes; a decreased function group that showed decreased visualization of lymphatic channels or delayed lymphatic flow, and an obstruction group that showed abnormal dermal backflow or few or no axillary lymph nodes. The authors also measured arm circumference at five standardized levels, and calculated the maximal circumference differences (MCDs) in the most symptomatic area. After this, they went on to correlate these results with the results of quantitative analyses calculated by quantitative asymmetry index (QAI) in both arms in three ROIs per arm, respectively—one circular ROI for axillary lymph nodes and two rectangular ROIs for the upper arm and the forearm region, excluding the elbow and hand. The results show a direct correlation between QAI and qualitative patterns. In fact, patients with obstructive patterns showed lower QAI in the axillary lymph node ROI and higher QAI in the upper limb ROI. MCD also appears to be inversely related to the QAI of the axillary lymph node ROI. Regarding the decreased function pattern, axillary QAI is found to be reduced, and is the lowest amongst all measures, even compared to the normal pattern in the QAI of upper limb ROI. All this explains the reduced function of the lymphatic system with reduced axillary flow through the remaining lymph nodes and increased flow due to new lymphatic collaterals in the upper arm [26]. 

Kim P. et al. evaluated the use of the quantitative method in 201 patients without lymphedema after unilateral breast cancer surgery. They observed a higher probability of developing lymphedema (OR = 0.14, CI = 0.04–0.46) in patients presenting abnormal ratios of radioactivity between affected arms and normal axilla (RRA) [27].

Szuba A. and Strauss W. [28] enrolled 90 patients with lymphedema after breast cancer therapy. The severity of lymphedema was evaluated by lymphoscintigraphy with the quantification of RRA. Patients were re-evaluated after the completion of therapy for lymphedema. The authors concluded that there is a correlation between the ARR and the percentage reduction in edema volume after therapy.

Newly available software permits the extraction of new quantitative parameters. A recent example has been proposed by Keramida G. et al. The lymphatic drainage efficiency (LDE) measures the percentage of injected activity accumulating in ilio-inguinal nodes, and has shown potential value in clinical research [29].

All these studies show that the quantitative approach should be systematically placed side by side with the classical qualitative protocol, because it effectively illustrates lymphedema severity, and it may be of use in negative cases determined using qualitative methods.

At last, lymphoscintigraphy appears to be the best method for the evaluation of primary and secondary lymphedema with low radiation doses (about 1 mSv), high sensitivity, and even higher specificity.

Lymphoscintigraphy is not only useful for the diagnosis and staging of lymphedema severity, but also for the prediction of worsening or improvements after therapy. These abilities are displayed whether using the qualitative or the quantitative approach, although the latter is less diffuse, especially in the case of bilateral lymphedema. Quantitative lymphoscintigraphy should be seriously considered for implementation, but the lack of widely accepted protocol makes it difficult to introduce this method into clinical practice.

## 3. New Emerging Techniques for Lymphedema Assessment in Real Clinical Practice

Over the past two decades, new techniques have emerged for the study of the lymphatic system, and particularly lymphedematous pathology. One of these is indocyanine green fluorescence lymphangiography, which has better spatial resolution than classical lymphoscintigraphy but a limitation in terms of anatomic coverage, including limited depth in skin studies.

More recently, magnetic resonance lymphangiography (MRL) has been developed for lymphedema screening and follow-up. This has been made possible by new MRI protocols both with and without contrast, achieving incredible anatomical detail thanks to high-field machines. Noninvasive MRL (NIMRL), using heavily T2-weighted sequences with very long TR/TE, has the advantages of reducing the method time, eliminating the radioactive dose given to the patient and clinicians, and not using contrast agents, avoiding adverse reactions [30].

Also, in contrast-enhanced MRI, new contrast agents are being investigated that have been specifically created for MRL, such as Ultrasmall Superparamagnetic Iron Oxide Nanoparticles (USPIO) that are selectively captured in the lymphatic system and lymph nodes [30]; however, today, MR lymphoangiography is most frequently performed via the subcutaneous injection of gadolinium-based contrast agent. This provides high spatial resolution 3D imaging of the lymphatic vessels, thus selecting patients who can benefit from surgical treatments such as LVA and helping surgeons to study lymphatic structures in the anatomic region of interest [31]. A detailed description of the imaging protocols of the various MRL techniques is given in the article by Guerrini S. et al. [32].

In addition to allowing the accurate visualization of lymphatic structures, MRL allows us to measure qualitative parameters to better differentiate the severity of lymphedematous pathology. These parameters include the numbers of lymphatic vessels, estimated vessels’ diameters and signal, and the enhancement characteristics.

MRL can also assess alterations in flow dynamics, as proposed by Borri et al. [33] in a study that describes a five-parameter model that can predict flow velocity, and outlines the difference between healthy and affected arms (9.7 cm/min in the unaffected arm vs. 2.1 cm/min in the affected arm) as an additional quantitative parameter to stratify patients who are candidates for different treatment approaches.

Recently, Kim G. [34] proposed a new MRI-based staging system. Although validated on a small number of patients (45) with secondary lymphedema in the upper limb, this staging method is proposed as an accurate non-invasive marker for therapeutic planning. The grading system is based on an evaluation of STIR-weighted images on axial sections at three levels: elbow, 5–8 cm proximal to the radius head, and 5–8 cm distal from the olecranon. Here, on the three sections, the percentage of circumferential subcutaneous fluid infiltration was assessed and graduated. Stage 0 was assigned when no subcutaneous tissue infiltration was present at any level, stage 1 was given when circumferential infiltration did not exceed 50% in any section, stage 2 was when the circumferential fluid infiltration was greater than 50% in any of the sections, and finally stage 3 was given when all three sections demonstrated circumferential fluid infiltration greater than 75%. All patients were evaluated with ICG lymphography, lymphoscintigraphy, the Lymphedema Quality of Life Questionnaire (LYMQOL), International Society of Lymphology (ISL) staging and quantitative measurements (limb volume and L-Dex^®^). The study showed a strong correlation between advanced stages under MRI and those under ISL, as well as with dermal backflow shown by lymphoscintigraphy. A correlation was also shown between advanced stages under MRI and abnormal ICG lymphography patterns, larger percentage differences in limb volume and higher L-Dex^®^ ratios. Therefore, this staging method showed encouraging results when used in MRI-based evaluations, with excellent interpretive reproducibility and correlation with other methods, in addition to the use of no contrast agent, but with all the advantages that it provides. Certainly, validation on a larger number of patients is needed. Other sequences (also with contrast agents) could be evaluated to show greater correlation, even in early stages of lymphedema.

In recent years, the MRI method used for the diagnosis and stratification of lymphedema is taking on an increasing role, and at the same time, efforts have been made to increase its sensitivity by combining it with a complementary method, such as indocyanine green (ICG) fluorescent lymphography.

ICG fluorescence lymphography is a relatively recent and very promising method, especially when used in the early stages of lymphedema or when combined with other methods. This method, which was initially develop to identify the sentinel lymph node, has not only been evaluated as a staging modality, but also been used to assess candidacy for surgical intervention in lymphedema. The technique is based on the use of contrast agents such as indocynine green, exploiting their ability to absorb light at a certain wavelength in the near-infrared spectrum and simultaneously (in real-time) visualize its uptake and transit due to induced fluorescence through a dedicated camera. This method demonstrates a high spatial resolution that can allow for the precise localization of functional superficial lymphatic vessels, their transport capacity, any collateral lymphatic vessels, and the presence of dermal backflow that represents the pathological conditions of the underlying lymphatic vessels. Although this is a very sensitive method capable of demonstrating alterations in the lymphatic pathway even before volumetric changes, it has many disadvantages, the most important of which is the loss of visualization of vessels located more than 2 cm under the skin; in fact, the method is only able to study the most superficial lymphatic structures.

Various classifications have been proposed for the assessment of lymphedema severity, making it difficult to compare the ICG fluorescence method with others. One of the most widely used, and internationally accepted, is that of the group from the Department of Plastic Surgery of University of Texas M. D. Anderson Cancer Center, set out by authors Chang D., Suami H. and Skoracki R. [35]. The classification proposed by the authors came from a prospective study of 100 patients undergoing LVA. The aim was to evaluate not only the efficacy of surgical treatment at the various stages of lymphedema, but also to establish the role of ICG lymphography in the assessment and selection of patients. The MD Anderson lymphedema classification (MDAC) is divided into five stages based on the descriptive features of ICG lymphography. Stage 0: Many patent lymphatic vessels, no dermal backflow, normal contractility. Stage 1: Many patent lymphatic vessels, minimal, patchy dermal backflow, slightly delayed contractility. Stage 2: Moderate patent lymphatic vessels, segmental dermal backflow, moderately delayed contractility. Stage 3: Few patent lymphatic vessels, extensive dermal backflow involving the entire arm, minimal contractility. Stage 4: No patent lymphatic vessels, severe dermal backflow in the entire extremity and dorsum extending to the digits (finger/toe sign) and volar (palm/sole sign), no contractility. Stage 5: No patent lymphatic vessels, no dye movement, no contractility. The MDACC classification is reported in Table 3.

In the end, this study demonstrated the efficacy of LVA in reducing the severity of lymphedema; it was more effective in early stages and in upper extremities. Lymphography with ICG showed good results in defining the functional severity of lymphedema, and it helps surgeons to select patients for LVA. Finally, even in postoperative evaluation, lymphography with ICG could be useful when assessing changes in lymphedema status.

There is great concordance between the ICG fluorescence and lymphoscintigraphic methods, as shown in a recent study by Akita S. et al. [37]. The authors compared ICG to lymphoscintigraphy when used in 169 extremities with lymphedema after lymph node dissection, demonstrating sensitivity, specificity and accuracy of 97%, 54% and 81%, respectively. The authors reported a sensitivity of ICG of 97%, a specificity of 92% and an accuracy of 95% in patients in the early stage, and discordant findings were revealed between the two techniques. According to these findings, they suggest the use of ICG as the first procedure.

The knowledge of the lymphatic system’s anatomy and its change after surgery is essential, and it is crucial to recognize regenerative lymphatic collaterals in order to explain lymphedema severity. ICG lymphography appears to be effective in this context.

Suami H. et al. proposed that the mechanism of lymphatic drainage is an additional factor in determining the degree of severity of lymphedema. These authors, in a recent study [38], reviewed a series of images obtained by lymphoscintigraphy and ICG lymphography in patients undergoing lymph node dissection. They proposed three types of possible mechanisms of afferent lymphatic vessel regrowth: new lymphatic vessels, dermal backflow, or a combination of these. They suppose that the mechanism of regeneration proceeding through dermal backflow is associated with more severe lymphedema because of the smaller size of lymphatic capillaries.

The combined usage of ICG and MRL represents an opportunity to achieve maximum benefits in accurate preoperative studies on patients who are candidates for LVA.

MRI is, in fact, able to depict deep structures not visible to ICG lymphography, and is used for initial evaluations of the patient. This approach was proposed by Pons G. et al. [39] in a prospective study on 82 patients. They obtained a high rate of success in performing LVA thanks to the precise spatial localization achieved with MRL.

In recent years, contrast enhancement ultra sonography (CEUS), which has already shown potential use in sentinel lymph node mapping [40], has been considered for the preoperative evaluation of lymphatic vessels that are not detected by other techniques, such as ICG lymphography. CEUS uses lipid or protein microbubbles containing inert gases as contrast agents, but few studies have proven its efficacy in lymphedema.

A recent study by Jang S.et al. [41] evaluated, in 11 women with breast cancer treatment-related lymphedema (BCRL), whether CEUS can be used to identify target lymphatic vessels before LVA surgery. ICG lymphography was performed in 10 women and failed to identify any targets in 5 of them, but CEUS was able to identify all lymphatic channels. This shows how the use of CEUS may help surgeons with preoperative planning when ICG lymphography is inadequate. A single study is not enough to support the inclusion of CEUS in clinical practice, as it lacks standardized protocols. More prospective studies on a larger and more varied case series are therefore needed to evaluate the efficacy of CEUS, which, due to its low cost and availability, could bring numerous advantages.

### AI and Machine Learning: The Distant Future of Diagnostic Imaging

We are living through a period of technological innovation in which the symbiosis among devices, industries, software and clinicians is deeply entrenched. The most striking example is the development of AI^51^ and its increasing use in clinical practice. However, we are far from the “holy grail” of conscious diagnosis and the interpretation of images, in which context ethical and social problems will be opened up the likes of which humans have never faced before.

Medicine, particularly diagnostic imaging, as in many other fields, has been affected by the influence of artificial intelligence (AI), which finds applications from radiomics to machine learning based on modern neural networks. Radiomics is a quantitative approach to medical imaging, which aims at enhancing the existing data available to clinicians by means of advanced mathematical analyses [42]. We are still in the early days of this new “Copernican revolution” that is growing at an exponential rate, and is promising in the near future to assist physicians in identifying early pathological conditions that elude the human eye. Its other advantages include higher reproducibility rates, the absence of human faults such as loss of attention, and an increasing ability for self-learning; however, there is no shortage of disadvantages, considering that AI is still in its experimental stage, in which its potential applications should be regulated.

There are few studies demonstrating the real potential of applying AI in the context of lymphedema. An early example is a recent study by Son H. et al. [43], which showed the application potential of deep-learning (DL)-based algorithms for the early identification of lymphedema-induced fibrosis by computer tomography (CT). The study evaluated 27 patients with lymphedema by analyzing a total of 2138 CT cross-sectional images. Then, the results of the algorithms were compared, based on four indices, with those obtained from the two gold-standard methods for fibrosis identification, which are standardized circumference difference ratio (SCDR) and bioelectrical impedance (BEI). The results obtained showed good correlation with traditional methods, although the study shows many limitations.

In another study by Nowak S. et al. [44], the authors evaluated the effectiveness of a DL pipeline that can assess shape, volume, and asymmetry based on an MRI of the lower extremities of patients with lymphedema. The authors retrospectively evaluated 45 patients, obtaining results that will facilitate a standardized analysis of volume and tissue distribution, which could help in the diagnosis of lymphedema or its monitoring.

Deep learning algorithms can also help achieve reproducibility in operator-dependent methods, such as echography. In fact, the goal of a study by Goudarzi S. et al. was precisely to establish automatic segmentation within a dataset of 39 patients. This may, in the future, make the staging of BCRL more convenient and accessible [45].

The application of AI is not only limited to the field of diagnostic imaging, but, as far as lymphedema is concerned, it can be applied to geographic prevalence studies for forms of primary lymphedema secondary to filariasis; it here affects the field of rehabilitation by evaluating in real time the patient’s movements and guiding them better in their daily exercises, and is also relevant to the field of robotic surgery, and finally early detection through predictive patterns in image recognition. All these fields of application have been covered in a recent review by Elday A.S. et al. [46].

## 4. The Role of Imaging in the Prevention and Treatment of Lymphedema

Imaging is taking on an increasingly central role in patient assessments—not only for diagnostic purposes, as it was until a few decades ago, but also now in prevention and, when this is no longer feasible, in therapy. While the number of staging classifications of lymphedema has increased with the assessment of patients over time, the same has not happened for the imaging-related risk group classifications. This lack plays a key role in hindering the development of cancer-related lymphedema, because it makes it impossible to define an individual diagnostic–therapeutic protocol based on personal risk.

Of the predictive factors associated with an increased risk of developing lymphedema, most are related either to individual factors, such as weight and age, or to cancer-related factors such as stage, lymph node dissection, and radiation therapy. Thus, predictive factors related to imaging parameters are lacking, although some authors report an association with the dermal backflow sign. However, many consider dermal backflow to be a true early sign of lymphedema, even if it is clinically silent, rather than a predictive parameter.

The role of imaging in treating lymphedema has benefited from new techniques in the area of lymphatic vessel anatomization, as demonstrated by the use of MRL in the pre-operative setting; similarly, ICG lymphography has demonstrated its ability to precisely identify lymphatic vessels to be subjected to reconstructive surgery in real time. Nuclear medicine has shown great potential in adding pathophysiological data to anatomical imaging [47,48,49,50,51]. These advancements are of value in many clinical scenarios where scintigraphy guides the treatment, as demonstrated by radioguided surgery (RGS) [52,53].

## 5. Conclusions

Lymphedema still represents a largely underestimated disease with few effective therapeutic options. The main form of lymphedema is cancer-associated. Its prevention to date involves the avoidance of lymph node dissection in women without cancer invasion at this level. This is made possible by sentinel lymph node mapping, which can be used to depict the first draining lymph node in the anatomical region of the tumor. It is assumed that the absence of disease in the sentinel lymph node is an indication of a disease-free status in all other lymph nodes in the area. The evolution of techniques used to identify the sentinel lymph node has led to their use in the field of lymphedema. There is also a hybrid variant of sentinel lymph node mapping that is relatively novel and whose main goal is to prevent lymphedema as a consequence of axillary surgery. This method is called axillary reverse mapping (ARM) [54], and it aims to recognize, using ICG fluorescence, physiological arm drainage, and using the dual technique, the sentinel lymph node, in order to preserve axillary drainage during the dissection procedure, thus reducing the risk of developing lymphedema.

The main goals of imaging are early diagnosis, predicting the development of lymphedema in the future, correctly localizing structures of surgical interest, and predicting therapeutic outcomes [55]. Through the contribution of imaging, it is therefore possible to significantly reduce the incidence of post-treatment lymphedema, both in terms of the common presentation in the upper or lower limbs and in the more unusual presentations, such as genital lymphedema [56,57]. All this is possible thanks to the continuous evolution of existing techniques and the use of new ones [58,59,60,61]. One of the most promising methods is ICG lymphography, although, up to now, the role of lymphoscintigraphy has remained indisputable in diagnosis, staging, and predicting outcomes.

In conclusion, new studies on the role of function imaging that can define risk categories of lymphedema development are needed, just as it is necessary to define the role that imaging can play in the following noninvasive therapies, which can prevent, in some cases, advanced forms of lymphedema.

Table 4 lists the main advantages and disadvantages of the various imaging methods that currently exist for use in lymphedema.

## Figures and Tables

**Table 1:** Taiwan Lymphoscintigraphy Staging classification.

Category	Normal Lymphatic Drainage	Partial Obstruction			Total Obstruction		
Score	L-0	P-1	P-2	P-3	T-4	T-5	T-6
Proximal Lymph Node	+	+/↓	↓	-	-	-	-
Intermediate Lymph Node	-	-	+/-	+	-	-	-
Lymphatic Ducts	+	+/Distal	Distal/Engorged	-	Engorged/-	Engorged/-	-
Dermal Backflow	-	-	+ (Proximal/Distal)	+ (Distal/Entire)	+ (Distal)	+ (Entire)	-

From: Cheng MH, Pappalardo M, Lin C, Kuo CF, Lin CY, Chung KC. Validity of the novel Taiwan lymphoscintigraphy staging and correlation of Cheng lymphedema grading for unilateral extremity lymphedema. Ann Surg. 2018; 268:513–525 [19].

**Table 2:** Cheng’s Lymphedema Grading System.

Grade		Circumferential Difference (%)	Episodes of Cellulitis (Time/Year)	Taiwan Lymphoscintigraphy Staging	ICG Lymphography	Treatment
0		0–9	0–1	L-0, P-1, P-2	Patent lymphatic ducts	CDT LVA
I	IA	10–19	1–2	P-1, P-2, P-3	Patent lymphatic ducts	LVA
	IB			P-3, T-4	Diffuse dermal backflow	VLN transfer
II	IIA	20–29	2–3	P-1, P-2, P-3	Patent lymphatic ducts	LVA
	IIB			P-3, T-4, T-5	Diffuse dermal backflow	VLN transfer
III		30–39	3–4	P-3, T-4, T-5, T-6	Not performed	VLN transfer +additional procedures
IV		>40	>4	T-4, T-5, T-6	Not performed	VLN transfer +additional procedures

Abbreviations: CDT (complete decongestive therapy), ICG (indocyanine green lymphography), LVA (lymphovenous anastomosis), VLN (vascularized lymph node). From: Pappalardo M, Cheng M-H. Lymphoscintigraphy for the diagnosis of extremity lymphedema: Current controversies regarding protocol, interpretation, and clinical application. J Surg Oncol. 2020;121: 37–47. [18]

**Table 3:** MDACC classification.

Stage	0	1	2	3	4	5
Patent lymphatic vessels	++++	+++	++	+	0	0
Dermal backflow	0	1	++	+++	++++ finger/toe pal/signs	0
Lymphatic vessel contractility	++++	+++	++	+	0	0

From: Nguyen AT, Suami H, Hanasono MM, Womack VA, Wong FC, Chang EI. Long-term outcomes of the minimally invasive free vascularized omental lymphatic flap for the treatment of lymphedema. J Surg Oncol. 2017;115(1):84–89 [36].

**Table 4:** Methods Comparison.

	Advantages	Disadvantages	Ref.
Lymphoscintigraphy	Gold-standard method capable of assessing lymphodynamics and hallmark features such as dermal backflow underlying major staging systems. Can also define quantitative parameters such as transit time and amount of radiotracer accumulation and compare them with contralateral limb. Relatively low radiant dose (1 Sv), lower than other roentgendiagnostic methods.	Low spatial resolution in accurate identification of lymphatic vessels, even with SPECT-CT methods showing lower definition than other methods. Use of ionizing radiation and a dedicated nuclear medicine department. Relative high cost of radiotracer.	[58]
ICG lymphography	Real-time method with very high spatial resolution of the superficial lymphatic network that can accurately identify flow alterations such as the presence of dermal backflow. Ability to use the method in operative time to identify potential target vessels for LVA. Increased availability of the method due to small and portable NIR camera.	Inability to study lymphatic structures located more than 2–3 cm deep. High cost of contrast medium and operator-dependent method.	[59]
MRI lymphangiography	Best anatomical definition among methods of both superficial and deep lymphatic vessels essential for proper surgical planning. Possibility of not using contrast medium thanks to dedicated sequences such as STIR and DWI. Possibility to define quantitative parameters such as flow velocity and to integrate AI protocols.	Low availability of the method and possible artifacts as well as lack of standardized protocols.	[60]
Contrast enhancement ultrasound (CEUS)	Potential wide availability of the method with low cost. Transportability of the machine and possibility of real-time use during surgery.	Operator-dependent method with low spatial resolution with possibility of obtaining a limited number of parameters such as identification of large lymphatic vessels.	[61]

## References

[1] McLaughlin S.A., Wright M.J., Morris K.T., Giron G.L., Sampson M.R., Brockway J.P., Hurley K.E., Riedel E.R., Van Zee K.J. (2008). Prevalence of lymphedema in women with breast cancer 5 years after sentinel lymph node biopsy or axillary dissection: Objective measurements. J. Clin. Oncol..

[2] Petrek J.A., Senie R.T., Peters M., Rosen P.P. (2001). Lymphedema in a cohort of breast carcinoma survivors 20 years after diagnosis. Cancer.

[3] Segerstrom K., Bjerle P., Graffman S., Nystrom A. (1992). Factors that influ- ence the incidence of brachial oedema after treatment of breast cancer. Scand. J. Plast. Reconstr. Surg. Hand Surg..

[4] Committee E. (2016). The diagnosis and treatment of peripheral lymph- edema: 2016 consensus document of the International Society of Lymphology. Lymphology.

[5] Monticone M., Ferriero G., Keeley V., Brunati R., Liquori V., Maggioni S., Restelli M., Giordano A., Franchignoni F. (2022). Lymphedema quality of life questionnaire (LYMQOL): Cross-cultural adaptation and validation in Italian women with upper limb lymphedema after breast cancer. Disabil. Rehabil..

[6] Cuccurullo V., Rapa M., Catalfamo B., Cascini G.L. (2023). Role of Nuclear Sentinel Lymph Node Mapping Compared to New Alternative Imaging Methods. J. Pers. Med..

[7] Stout Gergich N.L., Pfalzer L.A., McGarvey C., Springer B., Gerber L.H., Soballe P. (2008). Preoperative assessment enables the early diagnosis and successful treatment of lymphedema. Cancer.

[8] Spiegel M., Vesti B., Shore A., Franzeck U.K., Becker F., Bollinger A. (1992). Pressure of lymphatic capillaries in human skin. Am. J. Physiol. Circ. Physiol..

[9] Vermeulen K., Vandamme M., Bormans G., Cleeren F. (2019). Design and Challenges of Radiopharmaceuticals. Semin. Nucl. Med..

[10] De Cicco C., Cremonesi M., Luini A., Bartolomei M., Grana C., Prisco G., Galimberti V., Calza P., Viale G., Veronesi U. (1998). Lymphoscintigraphy and Radioguided Biopsy of the Sentinel Axillary Node in Breast Cancer. J. Nucl. Med..

[11] Ballinger J.R. (2022). Challenges in Preparation of Albumin Nanoparticle-Based Radiopharmaceuticals. Molecules.

[12] Partsch H. (1995). Assessment of abnormal lymph drainage for the diagnosis of lymphedema by isotopic lymphangiography and by indirect lymphography. Clin. Dermatol..

[13] McNeill G.C., Witte M.H., Witte C.L., Williams W.H., Hall J.N., Patton D.D., Pond G.D., Woolfenden J.M. (1989). Whole-body lymphangioscintigraphy: Preferred method for initial assessment of the peripheral lymphatic system. Radiology.

[14] Szuba A., Shin W.S., Strauss H.W., Rockson S. (2003). The third circulation: Radionuclide lymphoscintigraphy in the evaluation of lymphedema. J. Nucl. Med..

[15] Villa G., Campisi C.C., Ryan M., Boccardo F., Di Summa P., Frascio M., Sambuceti G. (2019). Procedural Recommendations for Lymphoscintigraphy in the Diagnosis of Peripheral Lymphedema: The Genoa Protocol. Nucl. Med. Mol. Imaging.

[16] Hassanein A.H., Maclellan R.A., Grant F.D., Greene A.K. (2017). Diagnostic accuracy of lymphoscintigraphy for lymphedema and analysis of false negative tests. Plast. Reconstr. Surg.-Glob. Open.

[17] Gloviczki P., Calcagno D., Schirger A., Pairolero P.C., Cherry K.J., Hallett J.W., Wahner H.W. (1989). Noninvasive evaluation of the swollen extremity: Experiences with 190 lymphoscintigraphic examinations. J. Vasc. Surg..

[18] Pappalardo M., Cheng M.H. (2020). Lymphoscintigraphy for the diagnosis of extremity lymphedema: Current controversies regarding protocol, interpretation, and clinical application. J. Surg. Oncol..

[19] Cheng M.H., Pappalardo M., Lin C., Kuo C.F., Lin C.Y., Chung K.C. (2018). Validity of the novel Taiwan lymphoscintigraphy staging and correlation of cheng lymphedema grading for unilateral extremity lymphedema. Ann. Surg..

[20] Cheng M.H., Chang D.W., Patel K.M. (2016). Principles and Practice of Lymphedema Surgery.

[21] Campisi C.C., Ryan M., Villa G., Di Summa P., Cherubino M., Boccardo F., Campisi C. (2019). Rationale for study of the deep subfascial lymphatic vessels during lymphoscintigraphy for the diagnosis of peripheral lymphedema. Clin. Nucl. Med..

[22] Fujiyoshi T., Mikami T., Hashimoto K., Asano S., Adachi E., Kagimoto S., Yabuki Y., Kitayama S., Matsubara S., Maegawa J. (2021). Pathological changes in the lymphatic system of patients with secondary lower limb lymphedema based on single photon-emission computed tomography/computed tomography/lymphoscintigraphy images. Lymphat. Res. Biol..

[23] Sacks G.A., Sandler M.P., Born M.L., Claton J.A., Franklin J.D., Partain C.L. (1983). Lymphoscintigraphy as an adjunctive procedure in the perioperative assessment of patients undergoing microlymphaticovenous anastomoses. Clin. Nucl. Med..

[24] Kwon H.R., Hwang J.H., Mun G.-H., Hyun S.H., Moon S.H., Lee K.-H., Choi J.Y. (2021). Predictive role of lymphoscintigraphy undergoing lymphovenous anastomosis in patients with lower extremity lymphedema: A preliminary study. BMC Med. Imaging.

[25] Yoo J.N., Cheong Y.S., Min Y.S., Lee S.W., Park H.Y., Jung T.D. (2015). Validity of quantitative lymphoscintigraphy as a lymphedema assessment tool for patients with breast cancer. Ann. Rehabil. Med..

[26] Chiewvit S., Kumnerdnakta S. (2017). Lymphoscintigraphic Findings That Predict Favorable Outcome after Lymphaticovenous Anastomosis. Lymphology.

[27] Kim P., Lee J.K., Lim O.K., Park H.K., Park K.D. (2017). Quantitative Lymphoscintigraphy to Predict the Possibility of Lymphedema Development After Breast Cancer Surgery: Retrospective Clinical Study. Ann. Rehabil. Med..

[28] Szuba A., Strauss W., Sirsikar S.P., Rockson S.G. (2002). Quantitative radionuclide lymphoscintigraphy predicts outcome of manual lymphatic therapy in breast cancer-related lymphedema of the upper extremity. Nucl. Med. Commun..

[29] Keramida G., Wroe E., Winterman N., Aplin M., Peters A.M. (2018). Lymphatic drainage efficiency: A new parameter of lymphatic function. Acta Radiol..

[30] Bellin M.F., Beigelman C., Precetti-Morel S. (2000). Iron oxide-enhanced MR lymphography: Initial experience. Eur. J. Radiol..

[31] Neligan P.C., Kung T.A., Maki J.H. (2017). MR lymphangiography in the treatment of lymphedema. J. Surg. Oncol..

[32] Guerrini S., Gentili F., Mazzei F.G., Gennaro P., Volterrani L., Mazzei M.A. (2020). Magnetic resonance lymphangiography: With or without contrast?. Diagn. Interv. Radiol..

[33] Borri M., Schmidt M.A., Gordon K.D., Wallace T.A., Hughes J.C., Scurr E.D., Koh D.-M., Leach M.O., Mortimer P.S. (2015). Quantitative Contrast-Enhanced Magnetic Resonance Lymphangiography of the Upper Limbs in Breast Cancer Related Lymphedema: An Exploratory Study. Lymphat. Res. Biol..

[34] Kim G., Smith M.P., Donohoe K.J., Johnson A.R., Singhal D., Tsai L.L. (2020). MRI staging of upper extremity secondary lymphedema: Correlation with clinical measurements. Eur. Radiol..

[35] Chang D.W., Suami H., Skoracki R. (2013). A prospective analysis of 100 consecutive lymphovenous bypass cases for treatment of extremity lymphedema. Plast. Reconstr. Surg..

[36] Nguyen A.T., Suami H., Hanasono M.M., Womack V.A., Wong F.C., Chang E.I. (2016). Long-term outcomes of the minimally invasive free vascularized omental lymphatic flap for the treatment of lymphedema. J. Surg. Oncol..

[37] Akita S., Mitsukawa N., Kazama T., Kuriyama M., Kubota Y., Omori N., Koizumi T., Kosaka K., Uno T., Satoh K. (2013). Comparison of lymphoscintigraphy and indocyanine green lymphography for the diagnosis of extremity lymphoedema. J. Plast. Reconstr. Aesthetic Surg..

[38] Suami H., Koelmeyer L., Mackie H., Boyages J. (2018). Patterns of lymphatic drainage after axillary node dissection impact arm lymphoedema severity: A review of animal and clinical imaging studies. Surg. Oncol..

[39] Pons G., Clavero J.A., Alomar X., Rodríguez-Bauza E., Tom L.K., Masia J. (2019). Preoperative planning of lymphaticovenous anastomosis: The use of magnetic resonance lymphangiography as a complement to indocyanine green lymphography. J. Plast. Reconstr. Aesthetic Surg..

[40] Huang Y., Zheng S., Lin Y. (2022). Accuracy and Utility of Preoperative Ultrasound-Guided Axillary Lymph Node Biopsy for Invasive Breast Cancer: A Systematic Review and Meta-Analysis. Comput. Intell. Neurosci..

[41] Jang S., Lee C.U., Hesley G.K., Knudsen J.M., Brinkman N.J., Tran N.V. (2022). Lymphatic Mapping Using US Microbubbles before Lymphaticovenous Anastomosis Surgery for Lymphedema. Radiology.

[42] Panico A., Gatta G., Salvia A., Grezia G.D., Fico N., Cuccurullo V. (2023). Radiomics in Breast Imaging: Future Development. J. Pers. Med..

[43] Son H., Lee S., Kim K., Koo K.I., Hwang C.H. (2022). Deep learning-based quantitative estimation of lymphedema-induced fibrosis using three-dimensional computed tomography images. Sci. Rep..

[44] Nowak S., Henkel A., Theis M., Luetkens J., Geiger S., Sprinkart A.M., Pieper C.C., Attenberger U.I. (2023). Deep learning for standardized, MRI-based quantification of subcutaneous and subfascial tissue volume for patients with lipedema and lymphedema. Eur. Radiol..

[45] Goudarzi S., Whyte J., Boily M., Towers A., Kilgour R.D., Rivaz H. (2023). Segmentation of Arm Ultrasound Images in Breast Cancer-Related Lymphedema: A Database and Deep Learning Algorithm. IEEE Trans. Biomed. Eng..

[46] Eldaly A.S., Avila F.R., A Torres-Guzman R., Maita K., Garcia J.P., Serrano L.P., Forte A.J. (2022). Artificial intelligence and lymphedema: State of the art. J. Clin. Transl. Res..

[47] Briganti V., Cuccurullo V., Di Stasio G.D., Mansi L. (2019). Gamma emitters in pancreatic endocrine tumors imaging in the pet era: Is there a clinical space for ^99m^Tc-peptides?. Curr. Radiopharm..

[48] Cuccurullo V., Manti F., De Risi M., Cascini G.L. (2021). DG-CT/PET false positive case in hip prosthesis: A clue to avoid error. Radiol. Case Rep..

[49] Cuccurullo V., Di Stasio G.D., Manti F., Arcuri P., Damiano R., Cascini G.L. (2021). The Role of Molecular Imaging in a Muscle-Invasive Bladder Cancer Patient: A Narrative Review in the Era of Multimodality Treatment. Diagnostics.

[50] Parmeggiani D., Gambardella C., Patrone R., Polistena A., De Falco M., Ruggiero R., Cirocchi R., Sanguinetti A., Cuccurullo V., Accardo M. (2017). Radioguided thyroidectomy for follicular tumors: Multicentric experience. Int. J. Surg..

[51] Cuccurullo V., Di Stasio G.D., Mansi L. (2017). Radioguided surgery with radiolabeled somatostatin analogs: Not only in GEP-NETs. Nucl. Med. Rev..

[52] Cuccurullo V., Cioce F., Sica A., Gatta G., Rubini G. (2012). Gastroenteric diseases in the third millennium: A rational approach to optimal imaging technique and patient selection. Recent. Progress. Med..

[53] Cuccurullo V., Di Stasio G., Prisco M., Mansi L. (2017). Is there a clinical usefulness for radiolabeled somatostatin analogues beyond the consolidated role in NETs?. Indian J. Radiol. Imaging.

[54] Wijaya W.A., Peng J., He Y., Chen J., Cen Y. (2020). Clinical application of axillary reverse mapping in patients with breast cancer: A systematic review and meta-analysis. Breast.

[55] Rajpurkar P., Lungren M.P. (2023). The Current and Future State of AI Interpretation of Medical Images. N. Engl. J. Med..

[56] Ciudad P., Bolletta A., Kaciulyte J., Losco L., Manrique O.J., Cigna E., Mayer H.F., Escandón J.M. (2023). The breast cancer-related lymphedema multidisciplinary approach: Algorithm for conservative and multimodal surgical treatment. Microsurgery.

[57] Kaciulyte J., Garutti L., Spadoni D., Velazquez-Mujica J., Losco L., Ciudad P., Marcasciano M., Lo Torto F., Casella D., Ribuffo D. (2021). Genital Lymphedema and How to Deal with It: Pearls and Pitfalls from over 38 Years of Experience with Unusual Lymphatic System Impairment. Medicina.

[58] Forte A.J., Boczar D., Huayllani M.T., Lu X., Ciudad P. (2019). Lymphoscintigraphy for Evaluation of Lymphedema Treatment: A Systematic Review. Cureus.

[59] Yamamoto T., Yamamoto N. (2022). Indocyanine Green Lymphography for Evaluation of Breast Lymphedema Secondary to Breast Cancer Treatments. J. Reconstr. Microsurg..

[60] Salehi B.P., Sibley R.C., Friedman R., Kim G., Singhal D., Loening A.M., Tsai L.L. (2023). MRI of Lymphedema. J. Magn. Reason. Imaging.

[61] Lahtinen O., Vanninen R., Rautiainen S. (2022). Contrast-enhanced ultrasound: A new tool for imaging the superficial lymphatic vessels of the upper limb. Eur. Radiol. Exp..

